# Publisher Correction: Relative effects of land conversion and land-use intensity on terrestrial vertebrate diversity

**DOI:** 10.1038/s41467-022-29528-6

**Published:** 2022-03-30

**Authors:** Philipp Semenchuk, Christoph Plutzar, Thomas Kastner, Sarah Matej, Giorgio Bidoglio, Karl-Heinz Erb, Franz Essl, Helmut Haberl, Johannes Wessely, Fridolin Krausmann, Stefan Dullinger

**Affiliations:** 1 Department of Botany and Biodiversity Research, University of Vienna, Rennweg 14, 1030 Vienna, Austria; 2 Department of Economics and Social Sciences, Institute of Social Ecology, University of Natural Resources and Life Sciences, Vienna (BOKU), Schottenfeldgasse 29, 1070 Vienna, Austria; 3 Senckenberg Biodiversity and Climate Research Centre, Senckenberganlage 25, Frankfurt am Main, 60325 Germany

**Keywords:** Agroecology, Biodiversity, Biodiversity, Macroecology

Correction to: *Nature Communications* 10.1038/s41467-022-28245-4, published online 01 February 2022.

The Supplementary Software was inadvertently omitted from the original version of the published article. This has now been corrected in the HTML version of the Article.

## Supplementary information

Supplementary Software

