# Supplementary material for: Relative effects of land conversion and land-use intensity on terrestrial vertebrate diversity
Source: Nat Commun. 2022 Feb 1;13:615. doi: 10.1038/s41467-022-28245-4 (PMC8807604; doi:10.1038/s41467-022-28245-4)
Supplement: Supplementary file 1 — Supplementary Information [file 41467_2022_28245_MOESM1_ESM.pdf]

Supplementary Figures

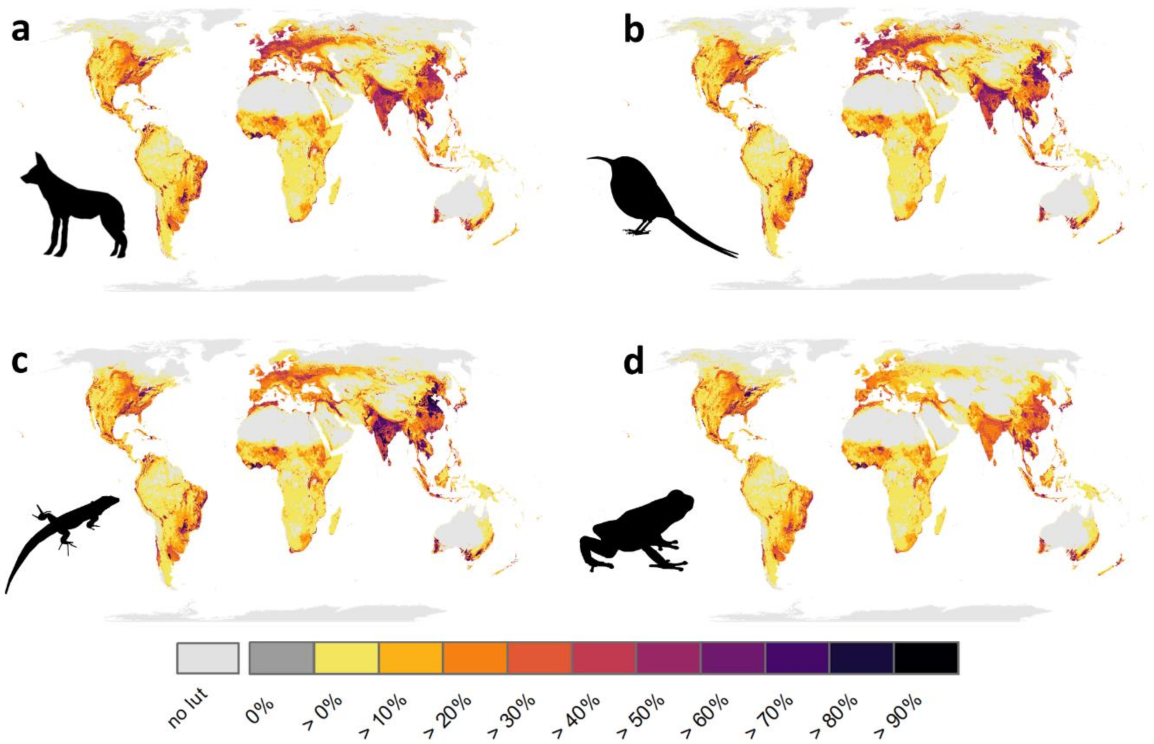

Supplementary Figure 1: Impending loss of terrestrial vertebrate species richness in response to current land-use activities*. Species losses are calculated separately for (a) mammals, (b) birds, (c) reptiles, and (d) amphibians. For more details see caption of Fig. 1 and Methods. No lut = no land-use type and simultaneous presence of indicated taxonomic group found within the respective landscape.*

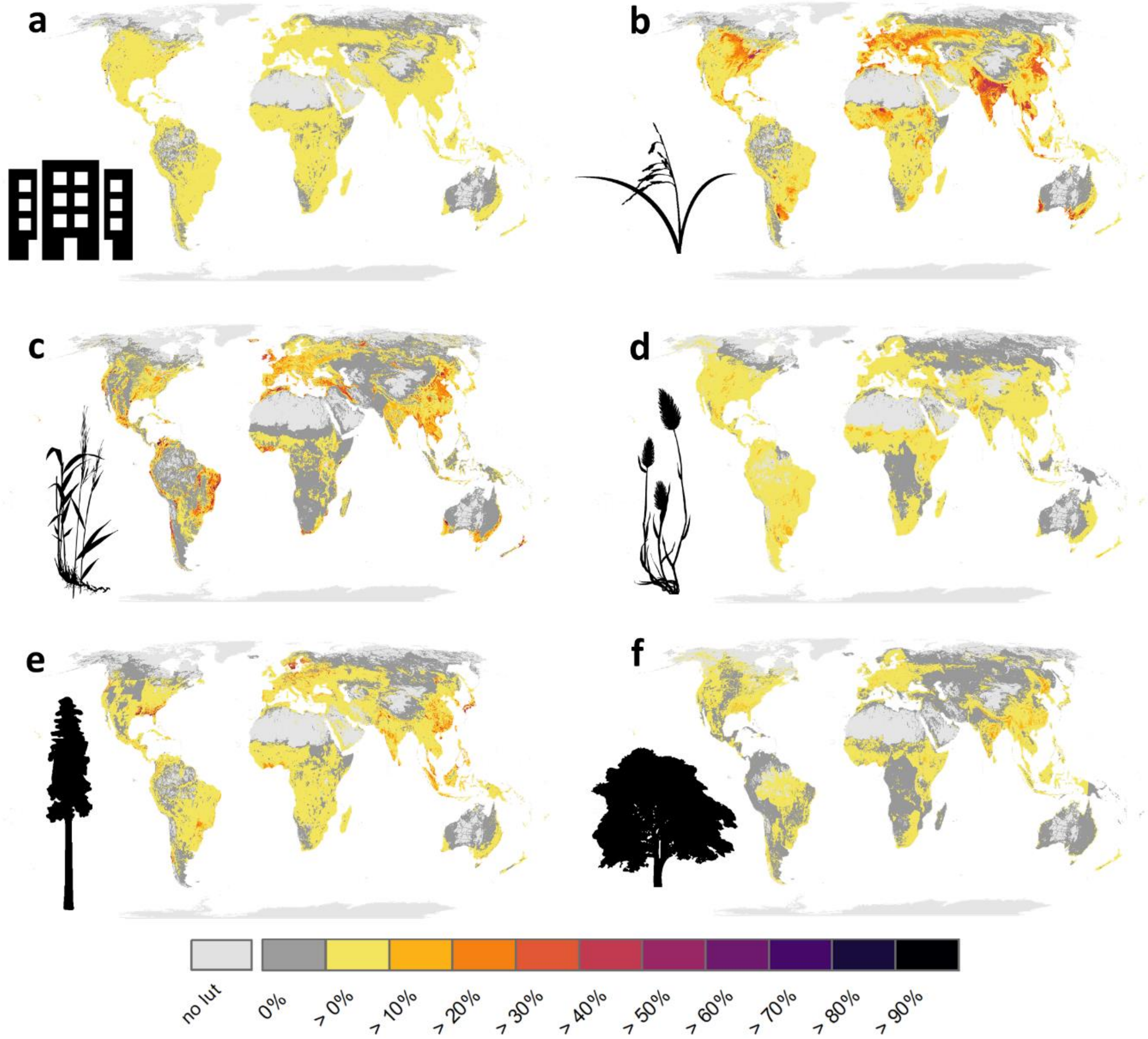

Supplementary Figure 2: Impending loss of terrestrial vertebrate species richness in response to current land-use activities*. Species losses are calculated separately for the aggregated broad land-use types (a) builtup, (b) cropland, (c) pastures, (d) grazing land, (e) plantations, and (f) forests. Numbers are proportions of all species of all taxonomic groups lost from a landscape due to the indicated land-use types. For more details see caption of Fig. 1 and Methods.*

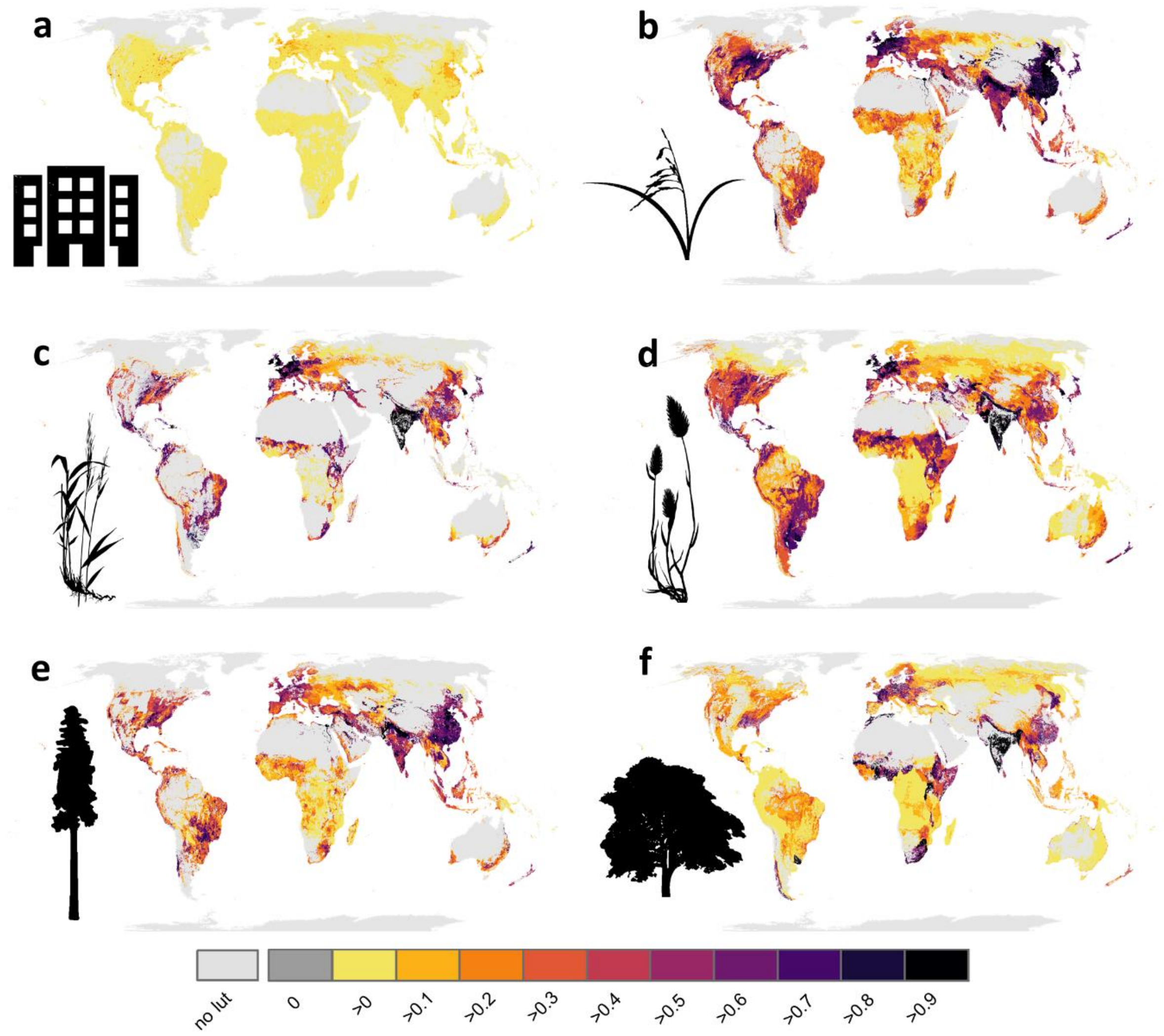

Supplementary Figure 3: Intensity of land-use per 5x5 arcmin landscape. *Shown for the land-use type aggregates (a) builtup, (b) cropland, (c) pastures, (d) grazing land, (e) plantations, and (f) forests. For cropland and plantations, the values are weighted means across all land-use types being aggregated into the respective broad land-use type. The weight of a land-use type corresponds to its proportional area within the landscape. Calculations were done separately for the two Sets of land-use-intensity indicators and averaged afterwards (Methods). No lut = the indicated land-use type is not present within the respective landscape.*

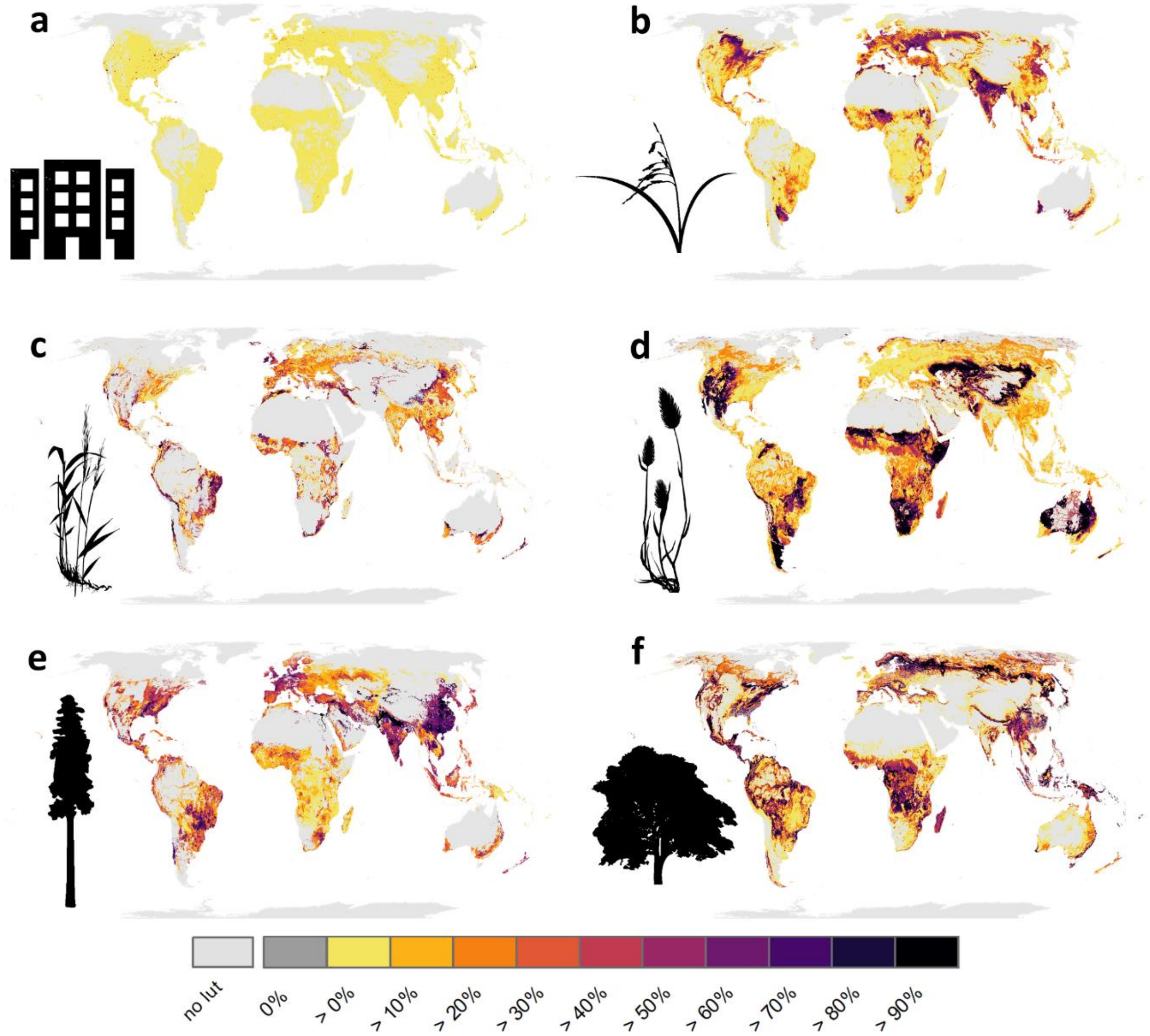

Supplementary Figure 4: Proportional area of each of the 6 land-use types per 5x5 arcmin landscape. *Shown for the land-use type aggregates (a) builtup, (b) cropland, (c) pastures, (d) grazing land, (e) plantations, and (f) forests. No lut = the indicated land-use type is not present within the respective landscape.*

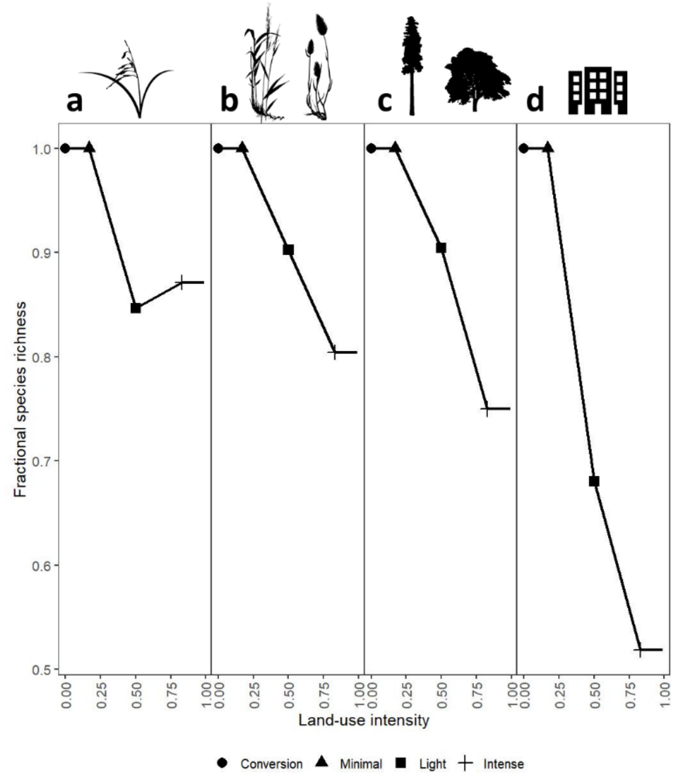

Supplementary Figure 5: Functions used to calculate the effect of land-use-intensity (*$\mathrm{LUI}_{n,b}$*) on species loss for land-use types (a) cropland, (b) pastures and grazing land, (c) forests and plantations, and (d) builtup*. More technically, the reduction of fractional species richness after conversion effects (*$\mathrm{f}_{n,b}$*, cf. Methods) have been accounted for. A fractional species richness of 1 represents the species richness under minimal use (equivalent to the category "Minimal" in ref[18], here defined as LUI=0.17) and includes all species whose area of habitat overlap with a focal landscape. In the case of the natural, unconverted land-use types grazing land and forests, fractional species richness is equal to the cell's native species richness (*$\mathrm{r}_{g,n,b}$*, Methods). Fractional species richness at the categorical LUI levels[18] 'Light' (here defined as LUI=0.5) and 'Intense' (here defined as LUI=0.83) represent the fractions of species remaining after intensification up to this level according to ref[5]. The lines between these categories represent linear interpolations between categorical levels.*

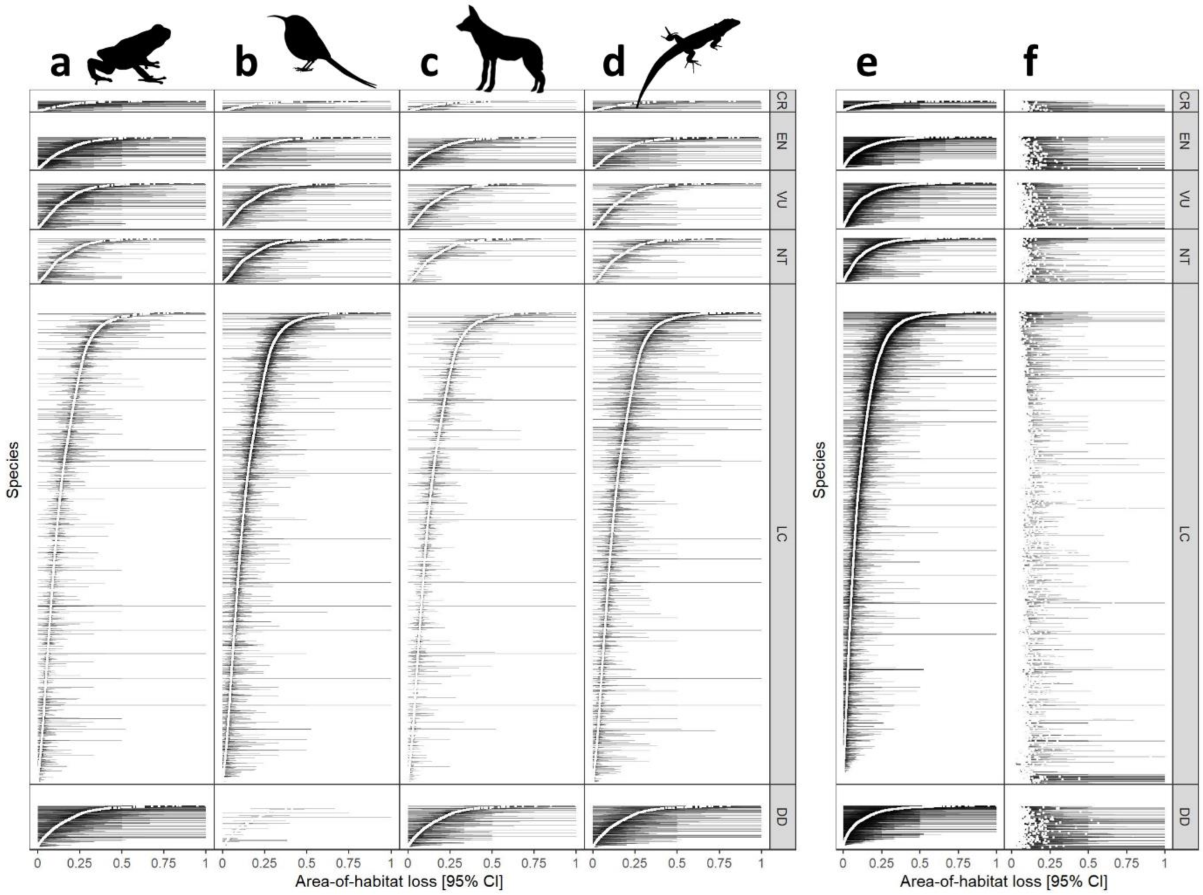

Supplementary Figure 6: Each species' calculated relative area-of-habitat loss caused by current land use for each taxonomic group (a-d) and caused by either conversion (e) or intensity (f)*. Shown are 95% confidence intervals and means (white dots). Each taxonomic group has different numbers of species, but the distance between lines are adjusted accordingly. The sequence of species in panel f follows the one in panel e, i.e. the mean (across 100 repeated calculations) proportional area of habitat loss they face from land conversion alone.*
