# Supplementary material for: Relative effects of land conversion and land-use intensity on terrestrial vertebrate diversity
Source: Nat Commun. 2022 Feb 1;13:615. doi: 10.1038/s41467-022-28245-4 (PMC8807604; doi:10.1038/s41467-022-28245-4)
Supplement: Supplementary file 3 — Description of Additional Supplementary Files [file 41467_2022_28245_MOESM3_ESM.docx]

**File Name: Supplementary Data 1
Description:** Summary statistics across all terrestrial 5x5 arcmin cells. Percentages impending species loss are averages across all cells weighted by cell area. Percentage of land area refer to the relative land area of all cells with more than the indicated relative SR  loss. For some summary statistics, both all available cells (incl. wilderness) and only cells where land-use is taking place (excl. wilderness) were used. For relative contributions of intensity, forests and grazing land were either included or excluded. Set 1 and Set 2 refer to the two different sets of intensity indicators used, see Methods for details.

**File Name: Supplementary Data 2**

**Description:** Translation, attribution and data sources of LU types and indicator sets across different levels within the cSAR framework. See Methods for details.

**File Name: Supplementary Data 3**

**Description:** Average native species’ area of habitat losses caused by current land use patterns worldwide decomposed into IUCN status, LU types or taxonomic group. Numbers are based on 100 random draws of selective species loss based on cSAR model results (Methods).

**File Name: Supplementary Data 4**

**Description:** Attribution of biomes to species’ habitat affiliations used to construct each species’ global area of habitat across 5x5 arcmin grid cell’s used to calculate species richness. See Methods for details.

**File Name: Supplementary Data 5**

**Description:** Summary statistics across all terrestrial 5x5 arcmin cells situated within a nation state. Percentages impending species loss are averages across all cells within the respective state weighted by cell area. Percentage of land area refer to the relative land area of all cells with more than the indicated relative SR  loss. Please note that these averages include only cells where land-use is taking place (excl. wilderness) and that they are based on the ensemble of two intensity indicator sets (Methods).

**File Name: Supplementary Data 6**

**Description:** Summary statistics across all terrestrial 5x5 arcmin cells situated within a biome as defined in Dinerstein et al. 2017. Percentages impending species loss are averages across all cells within the respective biome weighted by cell area. Percentage of land area refer to the relative land area of all cells with more than the indicated relative SR loss. Please note that these averages include only cells where land-use is taking place (excl. wilderness) and thatey are based on the ensemble of two intensity indicator sets (Methods).

**File Name: Supplementary Data 7**

**Description:** For the taxonomic group Amphibians, results of the random draw approach calculating each species’ area-of-habitat loss caused by global land-use practices. Given is the total area-of-habitat without land-use (in m^2^) and its proportional loss caused by land-use based on 100 random draws (see Methods for details). Median = median proportional loss, Mean = mean proportional loss, Lower = lower 95% confidence limit of proportional loss, Upper = upper 95% confidence limit of proportional loss.

**File Name: Supplementary Data 8**

**Description:** As in Supplementary Data 7 but for the taxonomic group Birds.

**File Name: Supplementary Data 9**

**Description:** As in Supplementary Data 7 but for the taxonomic group Mammals.

**File Name: Supplementary Data 10**

**Description:** As in Supplementary Data 7 but for the taxonomic group Reptiles.

**File Name: Supplementary Software**

**Description:** Contains R scripts (a) running the cSAR model based on the various input data (code_cSAR_model_NComms.r) and (b) running the random draw approach based on the cSAR model results (code_random_draw_NComms.r). More information on the functioning of each script, and the necessary input data, can be found within each script.
