# Supplementary material for: Relative effects of land conversion and land-use intensity on terrestrial vertebrate diversity
Source: Nat Commun. 2022 Feb 1;13:615. doi: 10.1038/s41467-022-28245-4 (PMC8807604; doi:10.1038/s41467-022-28245-4)
Supplement: Supplementary file 15 — Reporting Summary [file 41467_2022_28245_MOESM15_ESM.pdf]

nature portfolio

Corresponding author(s): Philipp Semenchuk
Last updated by author(s): Dec 7, 2021

Reporting Summary

Nature Portfolio wishes to improve the reproducibility of the work that we publish. This form provides structure for consistency and transparency in reporting. For further information on Nature Portfolio policies, see our Editorial Policies and the Editorial Policy Checklist.

Statistics

For all statistical analyses, confirm that the following items are present in the figure legend, table legend, main text, or Methods section.

n/a	Confirmed	
☒	☐	The exact sample size (n) for each experimental group/condition, given as a discrete number and unit of measurement
☒	☐	A statement on whether measurements were taken from distinct samples or whether the same sample was measured repeatedly
☐	☒	The statistical test(s) used AND whether they are one- or two-sided<br>*Only common tests should be described solely by name; describe more complex techniques in the Methods section.*
☐	☒	A description of all covariates tested
☐	☒	A description of any assumptions or corrections, such as tests of normality and adjustment for multiple comparisons
☐	☒	A full description of the statistical parameters including central tendency (e.g. means) or other basic estimates (e.g. regression coefficient) AND variation (e.g. standard deviation) or associated estimates of uncertainty (e.g. confidence intervals)
☐	☒	For null hypothesis testing, the test statistic (e.g. F, t, r) with confidence intervals, effect sizes, degrees of freedom and P value noted<br>*Give P values as exact values whenever suitable.*
☒	☐	For Bayesian analysis, information on the choice of priors and Markov chain Monte Carlo settings
☒	☐	For hierarchical and complex designs, identification of the appropriate level for tests and full reporting of outcomes
☒	☐	Estimates of effect sizes (e.g. Cohen's d, Pearson's r), indicating how they were calculated

Our web collection on statistics for biologists contains articles on many of the points above.

Software and code

Policy information about availability of computer code

Data collection	For collation, preparation and visualization of data, we used R v. 4.1.1 with the following packages: fasterize, rgdal, sf, data.table, stringr, pbapply, terra, rlist, tidyr, viridis, RColorBrewer
Data analysis	The R codes used to run the cSAR model and to calculate the native area-of-habitat loss of individual species are provided in the Supplementary Software file.

For manuscripts utilizing custom algorithms or software that are central to the research but not yet described in published literature, software must be made available to editors and reviewers. We strongly encourage code deposition in a community repository (e.g. GitHub). See the Nature Portfolio guidelines for submitting code & software for further information.

Data

Policy information about availability of data

All manuscripts must include a data availability statement. This statement should provide the following information, where applicable:

- Accession codes, unique identifiers, or web links for publicly available datasets
- A description of any restrictions on data availability
- For clinical datasets or third party data, please ensure that the statement adheres to our policy

The LU-intensity indicator Set 1 data generated in this study have been deposited in the Zenodo database under https://doi.org/10.5281/zenodo.5761990. These data are available under restricted access for reasons of separate future publication, access can be obtained by contacting the corresponding author to discuss the suitability of the data for the project in question and co-authorship of relevant authors.
The processed global land-use type distribution and area data processed in this study have been deposited in the Zenodo database under https://doi.org/10.5281/zenodo.5761990. These data are available under restricted access for reasons of separate future publication, access can be obtained by contacting the

corresponding author to discuss the suitability of the data for the project in question, co-authorship of relevant authors and details about the various datasets used to create these data.
The raw data generated in this study on species richness loss as displayed in Figs. 1 and 2, and Supplementary Figures 1 and 2 have been deposited in the Zenodo database under https://doi.org/10.5281/zenodo.5762083.
The area-of-habitat loss data of each species generated in this study, displayed in Figs. 3 and 4, and Supplementary Figure 6, are provided in the Supplementary Data files 7-10.
The range map and habitat affiliation data for amphibians, reptiles and mammals, and the elevational range data of all taxonomic groups used in this study are available in the IUCN Red List of Threatened Species database under https://www.iucnredlist.org/.
The range map data for birds used in this study are available in the BirdLife Bird species distribution maps of the world database and can be requested under http://datazone.birdlife.org/species/requestdis.
The biome (broad ecosystem types) data used in this study to define area-of-habitats are available in the Ecoregions 2017 database under https://ecoregions.appspot.com/.
The global elevation data used in this study are available in the GMTED2010 database under https://topotools.cr.usgs.gov/gmted_viewer/gmted2010_global_grids.php.
The nitrogen application rates data used in this study are available in the Earthstat database under http://www.earthstat.org/nutrient-application-major-crops/.
The gridded livestock information data used in this study are available in the FAO database under https://www.fao.org/livestock-systems/global-distributions/en/.

Field-specific reporting

Please select the one below that is the best fit for your research. If you are not sure, read the appropriate sections before making your selection.

☐ Life sciences ☐ Behavioural & social sciences ☒ Ecological, evolutionary & environmental sciences

For a reference copy of the document with all sections, see nature.com/documents/nr-reporting-summary-flat.pdf

Ecological, evolutionary & environmental sciences study design

All studies must disclose on these points even when the disclosure is negative.

Study description

We used the numerical cSAR model13 to calculate native species loss of four taxonomic groups (mammals, amphibians, reptiles, birds) caused by 45 LU types that were mapped onto a reference 5 x 5 arcmin grid (we also call individual grid cells landscapes in the following) of the global land area excluding Greenland and Antarctica. Calculations were based on (a) gridded LU-intensity and LU type information (see below), (b) effects of LU-intensity on species richness derived from recently published meta-analyses5,18, and (c) information on species distributions and habitat affiliations from IUCN and Birdlife International databases41,42. For presentation of results, we aggregated the calculated effects of the 45 LU types into those of six broad LU types (cropland (30 annual crop types); pastures (non-grassland converted to grassland); grazing land (natural/ near-natural areas with livestock grazing); builtup (sealed areas); plantations (11 permanent crop types plus timber plantations), and forests (natural/ near-natural forest under forestry); see Supplementary Data 2 for details).

Research sample

The LU-intensity indicator Set 1 data generated in this study have been deposited in the Zenodo database under https://doi.org/10.5281/zenodo.5761990. These data are available under restricted access for reasons of separate future publication, access can be obtained by contacting the corresponding author to discuss the suitability of the data for the project in question and co-authorship of relevant authors.
The processed global land-use type distribution and area data processed in this study have been deposited in the Zenodo database under https://doi.org/10.5281/zenodo.5761990. These data are available under restricted access for reasons of separate future publication, access can be obtained by contacting the corresponding author to discuss the suitability of the data for the project in question, co-authorship of relevant authors and details about the various datasets used to create these data.
The raw data generated in this study on species richness loss as displayed in Figs. 1 and 2, and Supplementary Figures 1 and 2 have been deposited in the Zenodo database under https://doi.org/10.5281/zenodo.5762083.
The area-of-habitat loss data of each species generated in this study, displayed in Figs. 3 and 4, and Supplementary Figure 6, are provided in the Supplementary Data files 7-10.
The range map and habitat affiliation data for amphibians, reptiles and mammals, and the elevational range data of all taxonomic groups used in this study are available in the IUCN Red List of Threatened Species database under https://www.iucnredlist.org/.
The range map data for birds used in this study are available in the BirdLife Bird species distribution maps of the world database and can be requested under http://datazone.birdlife.org/species/requestdis.
The biome (broad ecosystem types) data used in this study to define area-of-habitats are available in the Ecoregions 2017 database under https://ecoregions.appspot.com/.
The global elevation data used in this study are available in the GMTED2010 database under https://topotools.cr.usgs.gov/gmted_viewer/gmted2010_global_grids.php.
The nitrogen application rates data used in this study are available in the Earthstat database under http://www.earthstat.org/nutrient-application-major-crops/.
The gridded livestock information data used in this study are available in the FAO database under https://www.fao.org/livestock-systems/global-distributions/en/.

Sampling strategy

We selected all relevant datasets we were aware of. Within these datasets, no sampling was performed, i.e. we used the complete data.

Data collection

Data collection per se was not performed, i.e. no field work was conducted for this study. Instead, data were downloaded from the

	internet.
Timing and spatial scale	The data were downloaded once during the course of this study and largely consist of data covering the whole globe.
Data exclusions	No data were excluded.
Reproducibility	No experimental treatments were applied to the data. The calculation of all results can be verified by the data and code supplied as stated in the manuscript.
Randomization	We selected all data available, i.e. no sampling took place, and random selection from a given population was, hence, not performed.
Blinding	There were no questionnaires or other deciding tasks done in this study, therefore blinding was not necessary.

Did the study involve field work? ☐ Yes ☒ No

Reporting for specific materials, systems and methods

We require information from authors about some types of materials, experimental systems and methods used in many studies. Here, indicate whether each material, system or method listed is relevant to your study. If you are not sure if a list item applies to your research, read the appropriate section before selecting a response.

Materials & experimental systems

n/a	Involved in the study	
☒	☐	Antibodies
☒	☐	Eukaryotic cell lines
☒	☐	Palaeontology and archaeology
☒	☐	Animals and other organisms
☒	☐	Human research participants
☒	☐	Clinical data
☒	☐	Dual use research of concern

Methods

n/a	Involved in the study	
☒	☐	ChIP-seq
☒	☐	Flow cytometry
☒	☐	MRI-based neuroimaging
